# Linseed oil attenuates fatty liver disease in mice fed a high-carbohydrate diet

**DOI:** 10.1590/1414-431X2023e12927

**Published:** 2023-09-08

**Authors:** G. Godoy, C.C.O. Bernardo, L. Casagrande, M.L.M. Sérgio, J.N. Zanoni, J.V.C.M. Perles, R. Curi, R.B. Bazotte

**Affiliations:** 1 Programa de Pós-graduação em Ciências Farmacêuticas, Universidade Estadual de Maringá, Maringá, PR, Brasil; 2 Departamento de Ciências Morfológicas, Universidade Estadual de Maringá, Maringá, PR, Brasil; 3 Programa de Pós-graduação Interdisciplinar em Ciências da Saúde, Universidade Cruzeiro do Sul, São Paulo, SP, Brasil; 4 Seção de Produção de Imunobiológicos, Centro Bioindustrial, Instituto Butantan, São Paulo, SP, Brasil; 5 Departamento de Farmacologia e Terapêutica, Universidade Estadual de Maringá, Maringá, PR, Brasil

**Keywords:** Flaxseed oil, Omega 3 polyunsaturated fatty acids, Liver fibrosis, Hepatocellular ballooning, Hepatic steatosis, NAFLD

## Abstract

The impact of linseed oil as a lipid source on liver disease induced by a high-carbohydrate diet (HCD) was evaluated. Adult male Swiss mice received an HCD containing carbohydrates (72.1%), proteins (14.2%), and lipids (4.0%). The Control HCD group (HCD-C) received an HCD containing lard (3.6%) and soybean oil (0.4%) as lipid sources. The L10 and L100 groups received an HCD with 10 and 100% linseed oil as lipid sources, respectively. A group of mice were euthanized before receiving the diets (day 0) and the remaining groups after 56 days of receiving the diets (HCD-C, L10, and L-100 groups). Morphological and histopathological analyses, as well as collagen deposition were evaluated. Perivenous hepatocytes (PVH) of the HCD-C group were larger (P<0.05) than periportal hepatocytes (PPH) in the median lobe (ML) and left lobe (LL). There was a greater (P<0.05) deposition of type I collagen in PPH (*vs* PVH) and in the ML (*vs* LL). The ML exhibited a higher proportion of apoptotic bodies, inflammatory infiltrate, and hepatocellular ballooning. All these alterations (hepatocyte size, deposition of type I collagen, apoptotic bodies, inflammatory infiltrate, and hepatocellular ballooning) induced by HCD were prevented or attenuated in L10 and L100 groups. Another indicator of the beneficial effects of linseed oil was the lower (P<0.05) number of binucleated hepatocytes (HCD-C *vs* L10 or L100 group). In general, the L100 group had greater effects than the L10 group. In conclusion, linseed oil impedes or reduces the liver injury progression induced by an HCD.

## Introduction

Non-alcoholic fatty liver disease (NAFLD), defined by the excessive accumulation of fatty acids (FA) in the liver (1,2), represents the hepatic manifestation of a metabolic syndrome (3). The progression of NAFLD includes reversible lipid accumulation, non-reversible non-alcoholic steatohepatitis (NASH), which is associated with inflammation and discrete fibrosis process, and aggressive non-reversible cirrhosis, which is characterized by inflammation, consolidation of the fibrotic process, and liver failure (4). The investigation of NAFLD progression includes morphological evaluation, histopathological analysis, and quantification of biomarkers for inflammation and the fibrotic process (5).

High-carbohydrate diets (HCD) lead to the development of NAFLD by increasing FA accumulation in the liver (6). We have investigated the impact of an HCD on FA deposition and composition and inflammation in liver (7), skeletal muscle (8), brain (9), prefrontal cortex and hippocampus (10), and adipose tissue (11). Additionally, we have examined its effects on systemic inflammation (12,13). Our findings demonstrated that the replacement of conventional lipid sources with canola oil does not prevent HCD-promoted liver FA deposition and inflammation (14). Furthermore, we have reported that the myristic acid:docosahexaenoic acid ratio is a better biomarker of liver inflammation than the omega 6:omega 3 polyunsaturated FA (PUFA) ratio (15).

There is currently no specific treatment for NAFLD progression. The strategies employed include body weight loss and the administration of drugs that decrease insulin resistance (16-18). Linseed oil, a rich source of alpha-linolenic acid, has been reported to benefit cardiovascular diseases, renal diseases, diabetes, and NAFLD (19-22). For instance, Rezaei et al. (23) reported an association between linseed oil consumption and a reduction in liver FA in NAFLD patients. Linseed oil intake has also been shown to prevent NAFLD induced by a Western-type diet (24).

Recently, we demonstrated that increased inflammation, higher FA deposition, and increased lipid-occupied area in periportal hepatocytes (PPH) and perivenous hepatocytes (PVH) induced by HCD were prevented or attenuated by linseed oil as 10% (L10) or 100% (L100) of the lipid source (5). In this study, we expand upon these findings by assessing the impact of L10 and L100 on liver injury induced by HCD in PVH and PPH of the medium lobe (ML) and left lobe (LL). Additionally, new parameters of liver injury, including inflammation, ballooning, apoptosis, and fibrosis, were included in this study.

## Material and Methods

### Animals and experimental protocol

Male Swiss mice (*Mus musculus*) from the State University of Maringá Breeding Center (Brazil) were used. Mice weighing approximately 35 g (six weeks old) were housed in a room with controlled temperature (23±1°C), humidity (65±10%), and a 12-h light/dark cycle. The experimental protocol (3105210717) was approved by the Animal Ethics Committee of the State University of Maringá.

Before starting the experimental diets, all mice had free access to a standard rodent chow (QuimtiaTM, Brazil). After a 3-day of acclimatization period, the mice were randomly divided into three groups. The groups received a diet consisting of carbohydrates (72.1%), proteins (14.2%), and lipids (4.0%). The HCD-C (control) group received an HCD containing lard (3.6%) and soybean oil (0.4%) as lipid sources, the L10 group received an HCD containing lard (3.6%) and linseed oil (0.4%) as lipid sources, and the L100 group received an HCD containing only linseed oil (4.0%) as lipid source.

All diets were prepared using highly purified ingredients purchased from Rhoster^TM^ Company (Brazil). Lard was purchased from Sadia/BRF^TM^ (Brazil), soybean oil from Cocamar^TM^ (Brazil), and LO (*Linun usitatissimum*) from Vital Âtman^TM^ (Brazil).

Based on previous studies (5,7,11,14), we determined that 56 days of the diet is sufficient to induce NAFLD. This period also allows for the evaluation of the preventive or attenuating effects of L10 or L100 (5).

In addition to the HCD-C, L10, and L100 groups, we included a group of mice of the same age (six weeks old) as the other groups that was euthanized before initiating the experimental protocol (day 0). This fourth group served as a reference for the detrimental liver effects of HCD (7,11,14,22) and the potential beneficial impact of L10 and L100, as we have shown previously (5,22).

After 56 days of receiving the diets, the mice of the HCD, L10, and L100 groups were euthanized, and the median lobe (ML) and left lobe (LL) of the liver were sectioned and collected.

### Histological analysis

The ML and LL were washed in saline and then fixed in Bouin solution. After six hours, liver samples were dehydrated in a series of increasing alcohol concentration solutions, diaphanized in xylene, and embedded in paraffin. Slices were obtained using a microtome (Leica^TM^ RM2125 RTS, Germany), and semi-serial sections of 5-µm thickness were mounted on glass. Standard histological analysis was performed using hematoxylin and eosin staining, while Picrosirius staining was used for measuring type I and III collagen.

For the morphometric evaluation of the portal triad and centrilobular vein, images captured with a 40× objective were used to select the fields of PPH and PVH (10 hepatocytes per image, 10 images per mouse, 8-10 mice per group). Fields with only hepatocytes and no other structures were selected for hepatocyte quantification (10 images per mouse and 8-10 mice per group). The images were captured using the optical microscope Motic^TM^ BA 400 (Motic China Co, China) with a Moticam^TM^ 2500 5.0 Mega Pixel camera (Motic China Co). Image Pro-Plus^TM^ 4.0 software (Brazil) was used for morphometric analysis and quantification of hepatocytes.

**Figure 1:**
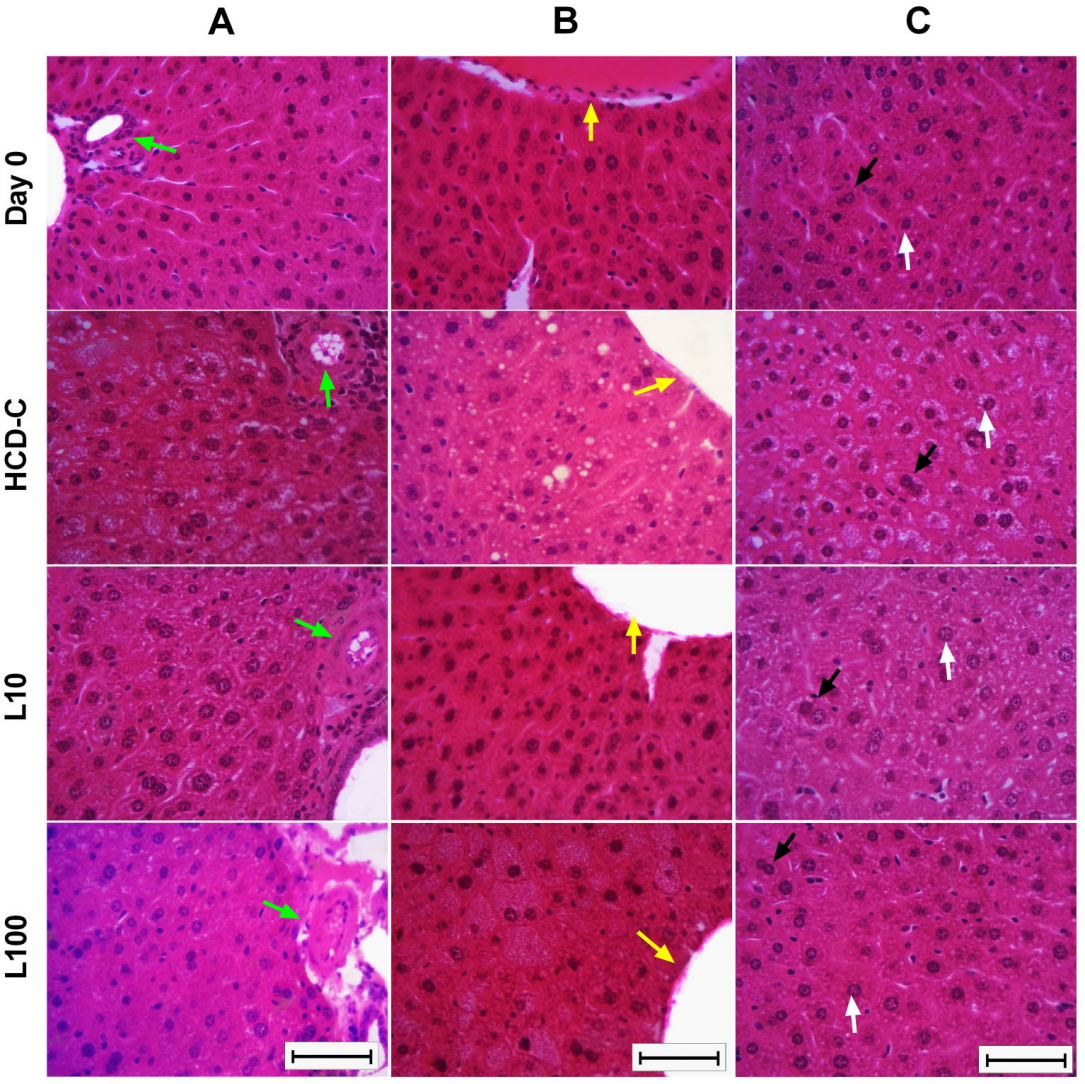
Representative photomicrographs of hematoxylin and eosin staining for quantification and morphometric analysis of hepatocytes obtained from periportal area (portal triad) (**A**), perivenous area (centrilobular vein) (**B**), and a clean field (**C**). The green arrows indicate the hepatic artery branch of the portal triad; the yellow arrows indicate the centrilobular vein branch; the white arrows indicate a mononucleated hepatocyte; the black arrows indicate a binucleated hepatocyte. The evaluations were done before (Day 0) and 56 days after starting the diets (high-carbohydrate diet-control (HCD-C), linseed 10% (L10), or linseed 100% (L100). The diet of each group is described in the Materials and Methods section. Scale bar: 50 µm.

For morphometric measurement of each image, the nuclear and cellular areas (µm^2^) were measured in hepatocytes with a visible cytoplasmic membrane, and then the cytoplasmic area (µm^2^) for each image was calculated using Image Pro-Plus^TM^ 4.0 software for each image. For hepatocyte quantification, a grid with 336 equidistant points 50 µm apart was overlaid on each image, and only mononucleated and binucleated hepatocytes with nuclei entirely within the grid were counted (Figure 1).

**Figure 2:**
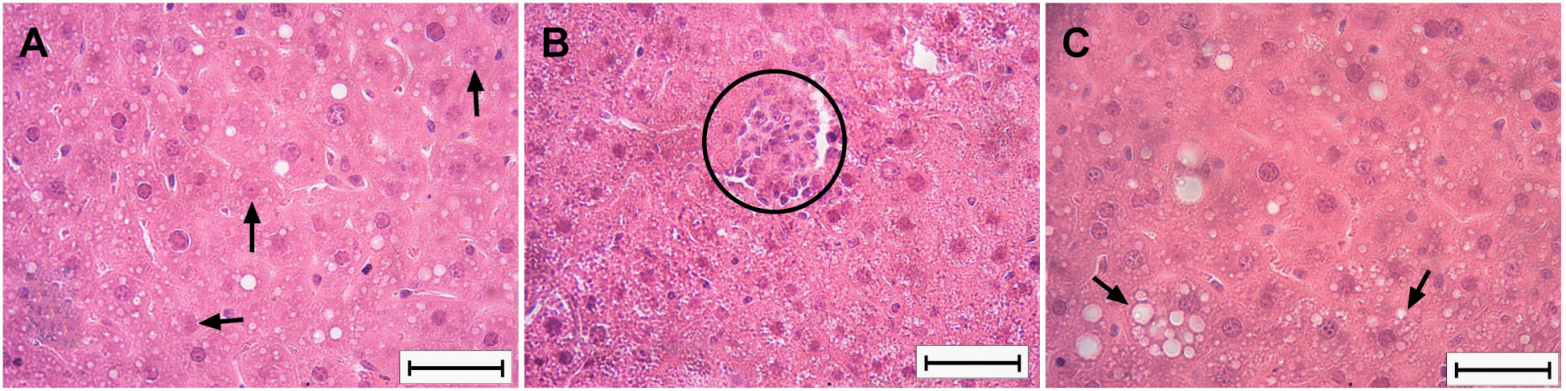
Representative photomicrographs of the histopathological analysis in hematoxylin and eosin staining in the liver. The black arrows indicate apoptotic bodies (**A**). The black circle marks inflammatory infiltrate (**B**). The black arrows indicate hepatocellular ballooning (**C**). Scale bar: 50 µm.

Histopathological analysis was performed on the same slices stained with hematoxylin and eosin under a 20× objective. The slices were divided into four quadrants, and the presence and intensity of the apoptotic bodies, inflammatory infiltrate, and hepatocellular ballooning were analyzed in each quadrant. Scores were assigned as follows: score 1: <5 fields, score 2: 6-10 fields, score 3: ≥11 fields; score 0: zero fields in the quadrant with apoptotic bodies, inflammatory infiltrate, and hepatocellular ballooning contents. The number of scores in the slice were summed, and then the slices were analyzed for each animal. The maximum score was 60 and the grade of apoptotic bodies, inflammatory infiltrate, and hepatocellular ballooning contents were classified as follows: 0-20 mild-grade; 21-40 moderate-grade; 41-60 severe-grade categories (Figure 2).

Collagen measurement was performed by Picrosirius staining in 5 slices from each mouse. Images were captured with a 20× objective on an Olympus^TM^ BX-53 optical microscope (Olympus Co., Japan) with a Roper Scientific Photometrics^TM^ digital camera (Roper Scientific, Germany) and an Olympus^TM^ light polarizer (Olympus Co.). The centrilobular vein or portal triad in the center (10 images per mouse, 8-10 mice per group) was used as a criterion for selecting the fields for comparison. Image Pro-Plus^TM^ 4.0 software (Media Cybernetics Inc., USA) was used to calculate the percentage of the area occupied by type I (red fibers) or type III collagen (green fibers) in each image, relative to the total size of the image.

### Statistical analysis

The data were analyzed using a block design followed by Fisher's test using Statistica^TM^ 8.0 software (StatSoft, Germany; https://www.statistica.com). Differences with P-values <0.05 were considered statistically significant.

## Results

### Morphometric analysis

The HCD-C group exhibited smaller (P<0.05) hepatocyte area, nuclei area, and cytoplasmic area in the PPH of ML and LL compared to Day 0 group (P<0.05). In addition, mice receiving HCD enriched with LO 10% (L10 group) or LO 100% (L100 group) had larger (P<0.05) hepatocyte area, nuclei area, and cytoplasmic area in the PPH of ML and LL (HCD-C group *vs* L-10 group or HCD-C group *vs* L-100 group). Furthermore, the L10 group had larger (P<0.05) hepatocyte area, nuclei area, and cytoplasmic area in the PPH of ML and LL compared to the L100 group (Table 1).

**Table 1:** Hepatocyte, nuclei, and cytoplasmic areas of periportal and perivenous hepatocytes.

Parameters	Periportal hepatocytes							
	Day 0 group		HCD-C group		L10 group		L100 group	
	ML	LL	ML	LL	ML	LL	ML	LL
Hepatocyte area (µm^2^)	110.88±0.82	105.18±0.83^d^	98.10±0.81^a^	96.05±1.04^a^	143.06±1.49^ab^	137.94±1.41^abd^	114.80±0.99^abc^	115.43±0.97^abc^
Nuclei area (µm^2^)	23.02±0.23	22.47±0.23	19.50±0.23^a^	21.04±0.30^ad^	28.409±0.34^ab^	25.99±0.32^abd^	22.87±0.26^bc^	22.65±0.25^bc^
Cytoplasmic area (µm^2^)	87.86±0.73	82.71±0.70^d^	78.61±0.69^a^	75.00±0.84^ad^	114.65±1.27^ab^	111.95±1.22^ab^	88.98±0.81^bc^	92.77±0.83^abcd^
	Perivenous hepatocytes							
	Day 0 group		HCD-C group		L10 group		L100 group	
	ML	LL	ML	LL	ML	LL	ML	LL
Hepatocyte area (µm^2^)	106.69±0.70	103.42±0.80^d^	121.21± 1.08^a^	134.39±1.47^ad^	121.25±1.00^a^	116.58±0.98^abd^	113.19±0.87^abc^	120.05±1.03^abcd^
Nuclei area (µm^2^)	24.17±0.25	24.05±0.27	24.99±0.31^a^	25.86±0.36^a^	25.71±0.31^a^	25.68±0.33^a^	25.84±0.29^abc^	25.02±0.29^ad^
Cytoplasmic area (µm^2^)	82.51±0.55	79.37±0.61^d^	96.21±0.87^a^	108.53±1.22^ad^	95.54±0.81^a^	90.89±0.74^abd^	87.35±0.71^abc^	95.03±0.83^abcd^

The evaluations were done before (Day 0) and 56 days after starting the diets (HCD-C: high-carbohydrate diet-control; L10: 10% linseed; L100: 100% linseed). The comparisons were done in the median lobe (ML) and left lobe (LL). Data are reported as means±SE of 8-10 mice. ^a^P<0.05 *vs* Day 0 in the same lobe; ^b^P<0.05 *vs* HCD-C in the same lobe; ^c^P<0.05 *vs* L10 in the same lobe; ^d^P<0.05 *vs* ML in the same group (ANOVA).

Mice receiving HCD enriched with 10% LO (L10 group) or 100% LO (L100 group) showed smaller (P<0.05) hepatocyte and cytoplasmic areas in the PVH of LL (HCD-C group *vs* L10 group or HCD-C group *vs* L100 group). For ML, a smaller (P<0.05) hepatocyte area and cytoplasmic area of PVH occurred only in the L100 group (HCD-C group *vs* L100 group). Additionally, the L10 group had higher (P<0.05) and lower (P<0.05) hepatocyte and cytoplasmic areas in the PVH of the ML and LL, respectively, than the L100 group (Table 1).

The results shown in the Table 1 were rearranged for a better understanding of the impact of HCD and the effect of LO incorporation in the HCD on PPH and PVH of the ML and LL.

On day 0, in the ML, the hepatocyte and cytoplasmic areas of the PPH were greater (P<0.05) than the PVH, whereas the nuclei area of the PVH was higher (P<0.05) than the PPH. Also, on day 0, in LL, the cytoplasmic area of the PPH was greater (P<0.05) than the PVH, whereas the nuclei area of the PPH was smaller (P<0.05) than the PVH (Table 2).

**Table 2:** Hepatocyte, nuclei, and cytoplasmic areas of cells from the median lobe and left lobe.

Parameters	Median lobe							
	Day 0 group		HCD-C group		L10 group		L100 group	
	PPH	PVH	PPH	PVH	PPH	PVH	PPH	PVH
Hepatocyte area (µm^2^)	110.88±0.82	106.69±0.70^d^	98.10±0.81	121.21± 1.08^d^	143.06±1.49	121.25±1.00^d^	114.80±0.99	113.19±0.87
Nuclei area (µm^2^)	23.02±0.23	24.17±0.25^d^	19.50±0.23	24.99±0.31^d^	28.409±0.34	25.71±0.31^d^	22.87±0.26	25.84±0.29^d^
Cytoplasmic area (µm^2^)	87.86±0.73	82.51±0.55^d^	78.61±0.69	96.21±0.87^d^	114.65±1.27	95.54±0.81^d^	88.98±0.81	87.35±0.71
	Left lobe							
	Day 0 group		HCD-C group		L10 group		L100 group	
	PPH	PVH	PPH	PVH	PPH	PVH	PPH	PVH
Hepatocyte area (µm^2^)	105.18±0.83	103.42±0.80	96.05±1.04	134.39±1.47^d^	137.94±1.41	116.58±0.98^d^	115.43±0.97	120.05±1.03^d^
Nuclei area (µm^2^)	22.47±0.23	24.05±0.27^d^	21.04±0.30	25.86±0.36^d^	25.99±0.32	25.68±0.33	22.65±0.25	25.02±0.29^d^
Cytoplasmic area (µm^2^)	82.71±0.70	79.37±0.61^d^	75.00±0.84	108.53±1.22^d^	111.95±1.22	90.89±0.74^d^	92.77±0.83	95.03±0.83^d^

The evaluations were done before (Day 0) and 56 days after starting the diets (HCD-C: high-carbohydrate diet-control; L10: 10% linseed; L100: 100% linseed). The comparisons were done between areas of periportal hepatocytes (PPH) and perivenous hepatocytes (PVH). Data are reported as means±SE of 8-10 mice. ^d^P<0.05 *vs* PPH in the same group (ANOVA).

The HCD group showed higher (P<0.05) hepatocyte area, nuclei area, and the cytoplasmic area in the PVH than the PPH of the ML and LL (Table 2).

The L10 group exhibited lower (P<0.05) hepatocyte, nuclei, and cytoplasmic areas of PVH than the PPH of the ML. In addition, the L10 group exhibited lower (P<0.05) hepatocyte and cytoplasmic areas of PVH compared to PPH of the LL (Table 2).

The L100 group showed a higher (P<0.05) nuclei area of PVH than PPH in the ML. Also, the L100 group had higher (P<0.05) hepatocyte area, nuclei area, and cytoplasmic area of PVH compared with PPH of the LL (Table 2).

### Hepatocyte quantification

The Day 0 group had a lower (P<0.05) amount of mononucleated hepatocytes compared to the HCD-C and L10 groups in the ML. Also, day 0 animals exhibited higher (P<0.05) amounts of binucleated hepatocytes than the L100 animals in the ML and LL.

The HCD-C group showed a higher (P<0.05) amount of mononucleated hepatocytes than the L100 group in the ML and LL. Additionally, the HCD-C group had a higher (P<0.05) amount of binucleated hepatocytes than the L10 and L100 groups the ML and LL.

The L10 group exhibited a higher (P<0.05) amount of mononucleated and binucleated hepatocytes compared to the L100 group in ML.

The L10 group had a higher (P<0.05) number of mononucleated hepatocytes in the ML than LL. Also, the Day 0, HCD-C, and L10 groups exhibited higher (P<0.05) amounts of binucleated hepatocytes in the ML than in LL (Table 3).

**Table 3:** Quantification of mononucleated and binucleated hepatocytes (cells/cm^2^) in the median lobe (ML) and left lobe (LL).

	Day 0 group		HCD-C group		L10 group		L100 group	
	ML	LL	ML	LL	ML	LL	ML	LL
Mononucleated cells	254.1±7.21	252.7±6.85	276.4±7.56^a^	262.7±7.73	292.3±8.79^a^	254.5±5.98^d^	245.3±5.98^bc^	243.3±6.15^b^
Binucleated cells	230.4±7.56	198.9±9.31^d^	245.2±10.72	216.5±9.14^d^	215.1±8.08^b^	190.0±8.08^bd^	180.1±7.38^abc^	174.2±7.56^ab^

The evaluations were done before (Day 0) and 56 days after starting the diets (HCD-C: high-carbohydrate diet-control; L10: 10% linseed; L100: 100% linseed). ^a^P<0.05 *vs* Day 0 in the same lobe; ^b^P<0.05 *vs* HCD-C in the same lobe; ^c^P<0.05 *vs* L10 in the same lobe; ^d^P<0.05 *vs* ML in the same group (ANOVA).

### Histopathological analysis

The Day 0 group scored practically 100% mild-grade on all parameters in both the ML and LL (Table 4).

**Table 4:** Histopathological analysis of apoptotic bodies, inflammatory infiltrate, and hepatocellular ballooning in the median and left lobes.

Parameters	Median lobe			
	Groups	Mild-grade(score 0-20) (%)	Moderate-grade(score 21-40) (%)	Severe-grade(score 41-60) (%)
Apoptotic bodies	Day 0	100	0	0
	HCD-C	0	88	12
	L10	71	29	0
	L100	67	33	0
Inflammatory infiltrate	Day 0	100	0	0
	HCD-C	13	62	25
	L10	71	29	0
	L100	67	33	0
Hepatocellular ballooning	Day 0	100	0	0
	HCD-C	0	25	75
	L10	29	0	71
	L100	11	56	33
Left lobe				
Apoptotic bodies	Day 0	100	0	0
	HCD-C	38	50	12
	L10	44	56	0
	L100	80	20	0
Inflammatory infiltrate	Day 0	89	11	0
	HCD-C	13	87	0
	L10	56	44	0
	L100	80	20	0
Hepatocellular ballooning	Day 0	100	0	0
	HCD-C	0	63	37
	L10	0	66	34
	L100	10	50	40

The evaluations were done before (Day 0) and 56 days after starting the diets (HCD-C: high-carbohydrate diet-control; L10: 10% linseed; L100: 100% linseed).

The HCD-C presented more moderate-grade and severe-grade scores than the Day 0, L10, and L100 groups in the ML and LL. The ML generally had more moderate-grade and severe-grade scores compared to the LL.

Incorporating LO in the HCD reduced the proportion of apoptotic bodies, inflammatory infiltrate, and hepatocellular ballooning, and the score was moderate instead of severe in the ML and LL.

### Collagen measurements

**Figure 3:**
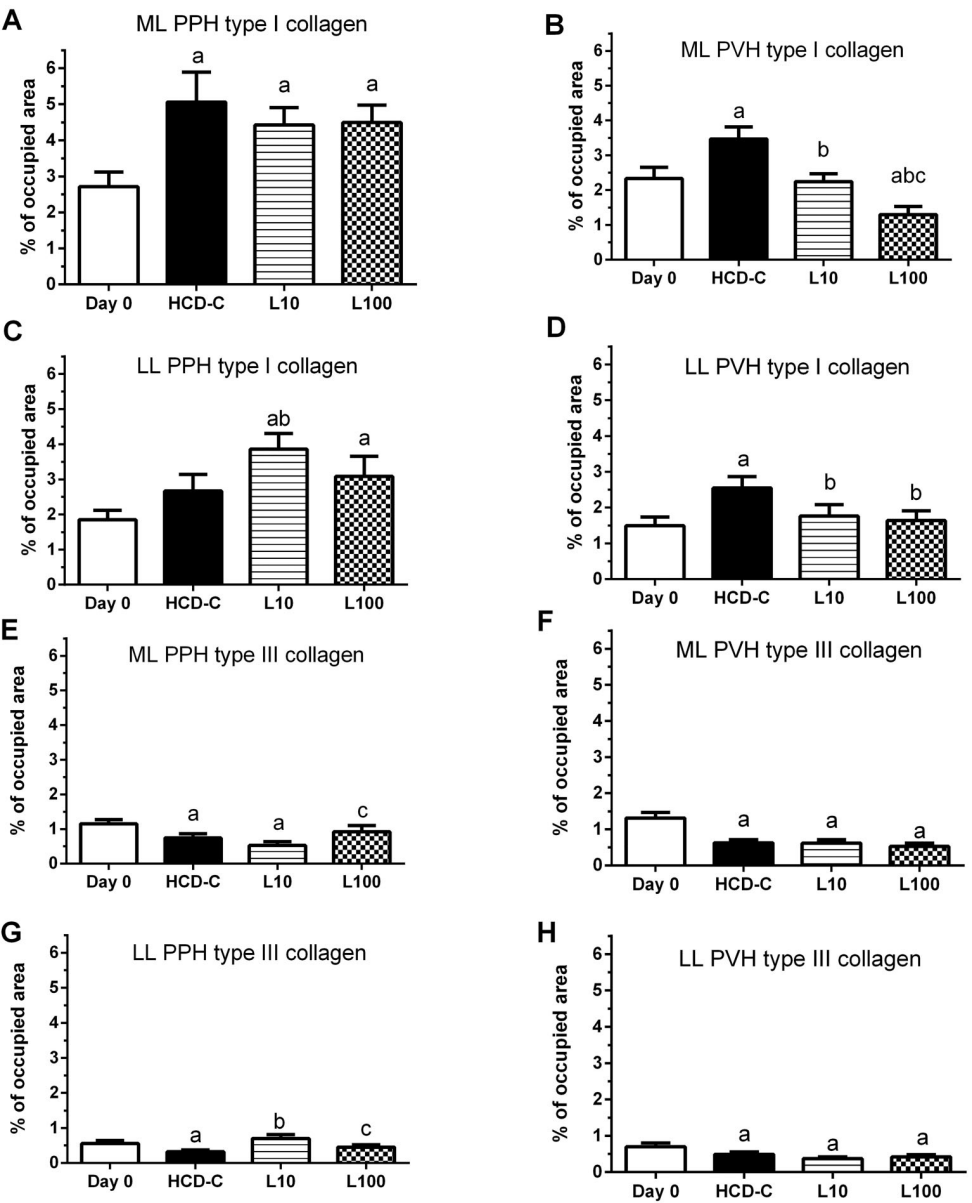
The histograms (**A**-**H**) represent the mean percent of occupied area by type I collagen and type III collagen in periportal hepatocytes (PPH) and perivenous hepatocytes (PVH) of the median lobe (ML) and left lobe (LL). The evaluations were done before (Day 0) and 56 days after starting the diets (high-carbohydrate diet-control (HCD-C), linseed 10% (L10), or linseed 100% (L100)). The diet of each group is described in the Materials and Methods section. Data are reported as mean and SD. ^a^P<0.05 *vs* Day 0; ^b^P<0.05 *vs* HCD-C (P<0.05); ^c^P<0.05 *vs* L10 (ANOVA).

Collagen measurements, reported as a percentage of the occupied area, are described in Figure 3, Table 5, and Table 6. Figure 3 shows the comparison between Day 0, HCD-C, L10, and L100 groups.

**Table 5:** Percentage of area occupied by type I collagen (red fibers) and type III collagen (green fibers) in the median lobe and left lobe.

Collagen	Median lobe							
	Day 0 group		HCD-C group		L10 group		L100 group	
	PPH	PVH	PPH	PVH	PPH	PVH	PPH	PVH
Red fibers	2.33±0.32	2.71±0.40	5.06±0.82	3.47±0.34*	4.42±0.48	2.24±0.22*	4.49±0.48	1.29±0.23*
Green fibers	1.15±0.11	1.31±0.15	0.74±0.11	0.62±0.08	0.52±0.10	0.61±0.09	0.92±0.17	0.52±0.09*
	Left lobe							
	Day 0 group		HCD-C group		L10 group		L100 group	
	PPH	PVH	PPH	PVH	PPH	PVH	PPH	PVH
Red fibers	1.84±0.27	1.49±0.24	2.66±0.47	2.54±0.32	3.85±0.44	1.76±0.32*	3.08±0.57	1.63±0.27*
Green fibers	0.55±0.08	0.70±0.10	0.32±0.05	0.49±0.06*	0.70±0.10	0.37±0.05*	0.45±0.06	0.41±0.06

The comparisons were done in the periportal hepatocytes (PPH) and perivenous hepatocytes (PVH) before (Day 0) and 56 days after starting the diets (HCD-C: high-carbohydrate diet-control; L10: 10% linseed; L100: 100% linseed). Data are reported as means±SE of 8-10 mice. *P<0.05 *vs* PPH in the same group (ANOVA).

**Table 6:** Percentage of area occupied by type I collagen (red fibers) and type III collagen (green fibers) in the periportal and perivenous areas.

Collagen	Periportal area							
	Day 0 group		HCD-C group		L10 group		L100 group	
	ML	LL	ML	LL	ML	LL	ML	LL
Red fibers	2.33±0.32	1.84±0.27*	5.06±0.82	2.66±0.47*	4.42±0.48	3.85±0.44	4.49±0.48	3.08±0.57*
Green fibers	1.15±0.11	0.55±0.08*	0.74±0.11	0.32±0.05*	0.52±0.10	0.70±0.10	0.92±0.17	0.45±0.06*
	Perivenous area							
	Day 0 group		HCD-C group		L10 group		L100 group	
	ML	LL	ML	LL	ML	LL	ML	LL
Red fibers	2.71±0.40	1.49±0.24*	3.47±0.34	2.54±0.32*	2.24±0.22	1.76±0.32*	1.29±0.23	1.63±0.27*
Green fibers	1.31±0.15	0.70±0.10*	0.62±0.08	0.49±0.06	0.61±0.09	0.37±0.05*	0.52±0.09	0.41±0.06

The comparisons were between the median lobe (ML) and left lobe (LL) before (Day 0) and 56 days after starting the diets (HCD-C: high-carbohydrate diet-control; L10: 10% linseed; L100: 100% linseed). Data are reported as means±SE of 8-10 mice. *P<0.05 *vs* median lobes in the same group (ANOVA).

For red fibers (type I collagen), the HCD-C, L10, and L100 groups showed a higher percentage (P<0.05) of occupied area compared to the Day 0 group in PPH area of the ML (Figure 3A). The HCD-C group exhibited a higher (P<0.05) percentage of the occupied area compared to the Day 0 group for red fibers in PVH area of the ML (Figure 3B) and LL (Figure 3D). The incorporation of LO in the HCD decreased (P<0.05) the percentage of the occupied area in PVH of the L10 (*vs* HCD-C) and L100 (*vs* HCD-C) groups of the ML (Figure 3B) and LL (Figure 3D). In contrast, the incorporation of LO in the HCD increased (P<0.05) the percentage of the occupied area in PPH of the LL in the L10 (*vs* HCD-C) (Figure 3C). For green fibers (type III collagen), the HCD-C, L10, and L100 groups showed a lower (P<0.05) percentage of occupied area compared to Day 0 group in PPH (Figure 3E) and PVH (Figure 3F) of the ML, and PVH of the LL (Figure 3H). In contrast, the incorporation of LO in the HCD increased (P<0.05) the percentage of occupied area in PPH of the LL in the L10 group (*vs* HCD-C group) (Figure 3G).

The results of the incorporation of LO in PPH and PPV of the ML and LL (Figure 3A-H) were rearranged in Tables 5 and 6. Table 5 shows the comparison between PPH and PVH, whereas Table 6 shows the comparison between the ML and LL.

For red fibers, the HCD-C, L10, and L100 groups showed a higher (P<0.05) percentage of the occupied area in PPH than in PVH in the ML. Also for red fibers, L10 and L100 groups showed a higher (P<0.05) percentage of the occupied area in PPH compared to PVH in the LL (Table 5).

For green fibers, the L10 and L00 groups exhibited a higher (P<0.05) percentage of the occupied area in PPH compared to PVH in the LL and ML, respectively. The HCD-C group showed a higher (P<0.05) percentage of the occupied area in PVH compared to the PPH in the LL (Table 5).

For red fibers, the Day 0, HCD-C, L10, and L100 groups showed a higher (P<0.05) percentage of the occupied area in the ML than the LL in the PPH and PPV. For green fibers, the Day 0, HCD-C, and L100 groups had a higher (P<0.05) percentage of the occupied area in the ML than LL in PPH. Also, the Day 0 and L10 groups showed a higher (P<0.05) percentage of the occupied area in the ML compared to LL in PVH (Table 6).

## Discussion

The liver is anatomically divided into lobes, each of which have functional structures known as lobules (25). Since the livers of mice are small and the anatomical division of lobes is not well-defined, we analyzed the ML and LL, which have a larger area, better anatomic position, and lobe division. Considering that liver lobes and lobules are complex structures, it is suggested that the complex intrahepatic vascular network and oxygen gradient are thought to promote the differentiation between PPH and PVH (26), and an HCD intensifies these differences. For example, PVH of the HCD-C group was larger (*vs* PPH) not only in the ML but also in the LL. These alterations were prevented or attenuated if the lipid source of the HCD (lard and soybean oil) was replaced by 10% (L10 group) or 100% (L100 group) linseed oil. Similary, our previous study using the same experimental model (5) demonstrated that the percentage of lipid-occupied area was higher in PVH, while the L10 diet and L100 diet prevented or decreased these alterations.

Liver fibrosis induced by the HCD was not homogeneous. There was a greater deposition of type I collagen in PPH (*vs* PVH), probably due to the presence of hepatic stellate cells (HSC) in this region (27). The activation of HSC increases collagen synthesis and promotes fibrosis progression (28-31). HSCs are activated by several factors, including increased lipid accumulation (32,33).

The ML from HCD-C mice exhibited a higher deposition of type I collagen compared to LL. Consistent with these results, the ML showed a higher proportion of apoptotic bodies, inflammatory infiltrate, and hepatocellular ballooning, while in general, linseed oil prevented or attenuated these changes. Apoptotic bodies resulting from the apoptotic process could be triggered by several factors, including FA (34). The inflammatory infiltrate is formed by the recruitment of immune cells during liver injury (35). The higher amount of liver inflammatory infiltrate in the HCD-C group and the lower levels in the animals that received linseed oil in the diet could be associated with the degree of FA deposition, which was higher in the HCD group than in the L10 and L100 groups (5). The mild-grade inflammatory infiltrate in the L100 group could be related to the anti-inflammatory properties of linseed oil (19-24). The presence of severe hepatocellular ballooning confirmed liver injury, which may be caused by the rearrangement of the cytoskeleton (36,37).

Another indicator of the beneficial effects of linseed oil was the lower number of binucleated hepatocytes, an indicator of liver injury (38), in the L10 and L100 groups compared to the HCD-C group.

The morphological and histopathological changes induced by the HCD and the effects of linseed oil showed different degrees of intensity depending on the parameter evaluated, hepatocyte zonation, or lobe localization. The heterogeneity of liver response to injury opens the possibility of evaluating liver diseases in the entire organ and the morphological and histopathological changes in specific lobes, lobules, and hepatocytes in the portal triad and centrilobular vein.

The L100 diet had more significant effects than the L10 diet. In agreement with this observation, we previously reported that the L100 diet, but not the L10 diet, prevented the elevation of blood triacylglycerol levels promoted by HCD (5).

Additionally, the L100 mice exhibited higher liver levels of omega-3 PUFA and lower levels of saturated FA, monounsaturated FA, and omega-6 PUFA compared to the L10 or HCD-C group, reflecting the higher proportion of omega-3 PUFA in the diet (5).

The beneficial effects of linseed oil demonstrated here and by others (39,40) were not influenced by changes in food intake and body weight (data not shown), as these parameters remained unchanged during the 56 days of experimental diets.

**Figure 4:**
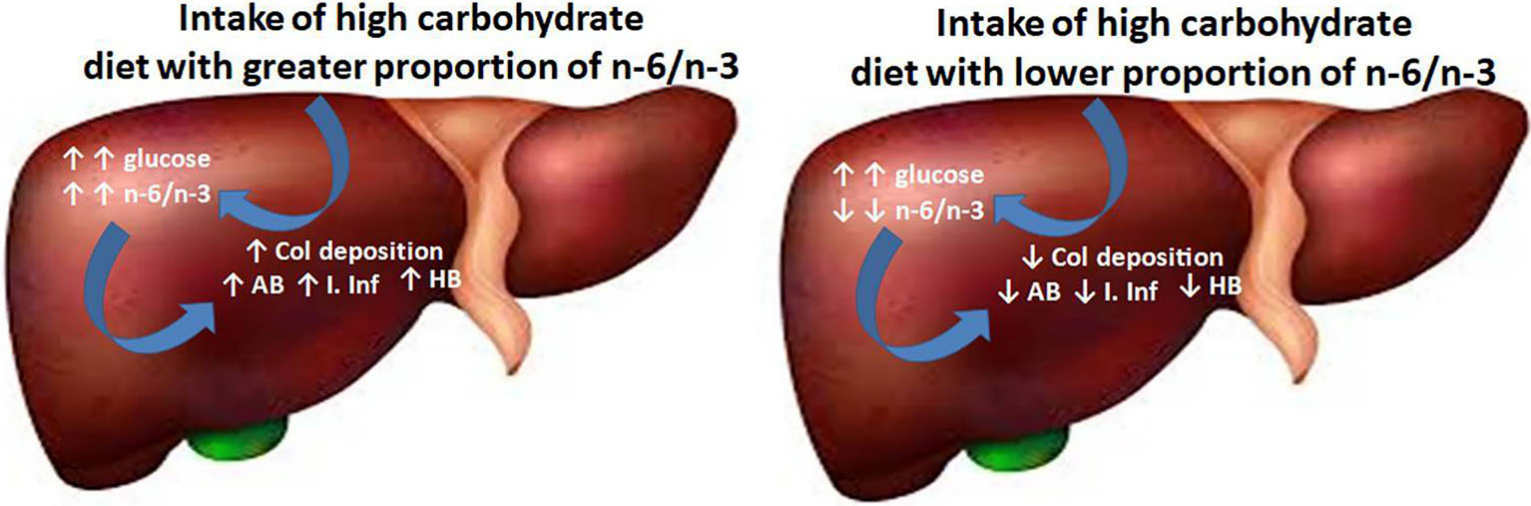
Comparative effects of a high carbohydrate diet (HCD) containing a greater proportion of omega-6:omega-3 polyunsaturated fatty acids (n-6/n-3) and an HCD containing a lower proportion of n-6/n-3. The lower proportion of n-6/n-3 reduces inflammatory infiltrate (I. Inf), collagen 1 (Col) deposition, percent of hepatocellular ballooning (HB), and apoptotic bodies (AB).

The results described are summarized in Figure 4.

In conclusion, ingestion of linseed oil prevents the progression of liver injury induced by an HCD. These results are relevant considering the absence of pharmacological approaches to prevent NAFLD progression and the difficulties in implementing a long-term weight loss, opening the possibility of using linseed oil as a coadjuvant to prevent or treat liver injury induced by an HCD.
